# Studying the Sintering Behavior of H_2_-Reduced Bauxite Residue Pellets Using High-Temperature Thermal Analysis

**DOI:** 10.3390/ma18102378

**Published:** 2025-05-20

**Authors:** Dali Hariswijaya, Jafar Safarian

**Affiliations:** Department of Material Science and Engineering, Faculty of Natural Sciences, Norwegian University of Science and Technology, 7034 Trondheim, Norway; jafar.safarian@ntnu.no

**Keywords:** bauxite residue, circular economy, hydrogen reduction, thermal analysis

## Abstract

Treating bauxite residue as an alternative source of metals for iron and aluminum industry is an approach to promote circular economy in metal industries. Reduction of metal oxides with a H_2_-based process is an important step for the decarbonization of metal industry. In this study, bauxite residue (BR) pellets were prepared and were reduced with different H_2_-H_2_O gas compositions at different temperatures, which yielded with various degrees of reduction. The bauxite residue pellets were made from a mixture of bauxite residue and Ca(OH)_2_ powders and sintered at 1150 °C. Hydrogen reduction was carried out on the oxide pellets using a resistance furnace at elevated temperatures in controlled reduction atmosphere of H_2_-H_2_O gas mixtures, which resulted in the reduction of iron oxides in the pellets. Unreduced and reduced pellets were subsequently heated to 1400 °C to study their sintering behavior during H_2_ reduction using differential thermal analysis (DTA) and thermogravimetric analysis (TGA) techniques to investigate the evolution of phases related to slag formation. Equilibrium module of Factsage™ was utilized to analyze results of thermal analysis. Both chemical and physical changes that occurred during the sintering process of the H_2_-reduced BR pellets were successfully detected by TG–DTA analysis, and the initial slag- and gas-phase formation were detected to occur from 900 °C and 1180 °C, respectively. One of the most notable chemical reactions to occur during the analysis was formation of mayenite at 810 °C, which is easily leachable, providing potential for recovery of alumina.

## 1. Introduction

### 1.1. Bayer Process and Bauxite Residue

The Bayer process was invented and patented by Carl Josef Bayer in 1888 and has since then been the leading process for alumina (Al_2_O_3_) production in the world. The process consists of eight main stages: milling, desilication, digestion, clarification, precipitation, evaporation, classification, and calcination. In the milling step, the bauxite ore is crushed down into finer particles. Additionally, limestone is added to create a pumpable slurry. After the milling step, the slurry moves through a process called desilication, which involves removing silica (SiO_2_). The slurry is then digested using a NaOH solution, which dissolves the aluminum-bearing minerals in the bauxite. These minerals include gibbsite (Al(OH)_3_, boehmite (γ-AlO(OH)), and diaspore (α-AlO(OH)) [1].

After the processing step, the slurry is cooled down using a series of flash tanks at 1 atm. The slurry is then prepared for clarification where the bauxite residue (BR) is separated away through sedimentation, where chemical additives assist in driving the BR to the bottom of the settling tanks. BR is transferred to washing tanks, where the goal is to recover the caustic soda used in the digestion step. The saturated liquid undergoes a series of filtration steps and BR is left in disposal areas. After clarification, alumina is recovered through crystallization during precipitation step [1].

Evaporation of the liquid used during crystallization takes place in heat exchangers, where it is subsequently cooled down afterwards in flash tanks. The condensate that is created through this process is re-used for BR washing or as feed water. Recovered caustic soda is then re-added to the digestion step. The crystals are classified into size ranges, using cyclones and gravity classification tanks. For the coarse crystals, separation from liquid and calcination is performed. For the finer crystals, washing to remove organic impurities and re-addition to the precipitation step is performed. Calcination of the coarse crystals is performed by roasting in calciners. The roasting process takes place at temperatures up to 1100 °C. This drives off moisture and water, which eventually creates alumina solids [1].

### 1.2. Reduction of Bauxite Residue

Red mud, also known as bauxite residue (BR) in dewatered form, is the main by-product generated in the Bayer process. Typically, for each ton of produced alumina from bauxite ore, about 1.5 tons of BR is produced [2]. The generated BR from the Bayer process is stored in large holding ponds, where only 1% to 2% is recycled [3]. Dried BR typically contains up to 50% of iron oxides [3]. Other compounds found in BR includes silica oxides, titanium oxides, aluminum oxides, and other oxides. It is also highly alkaline, with a pH level ranging from 12 to 13. Due to its high alkalinity, red mud that is stored away in holding ponds poses a great environmental threat to its surroundings. The main way of treating the alkaline BR is to attempt neutralizing by adding acidic substances, such as HCl [4].

Reduction of BR opens opportunities for recovery of its metal content, especially iron [5,6,7], aluminum [8,9], and rare-earth elements (REEs) [10]. It is to be understood from previous research that the removal of iron content is a necessary preliminary step for effective recovery of alumina and REEs in BR [8,10] and reduction is believed to be the most efficient method for removal of iron in BR. Several studies on reduction of BR have been conducted within the past decade, which mainly tries to recover iron content in BR either in the form of magnetite [8] or metallic iron [8,10,11,12,13].

Reduction of BR with H_2_ at low temperature (480 °C) was able to produce magnetite with 87% conversion degree with insignificant metallic iron production [14]. The addition of NaOH into BR pellets followed by solid–gas reduction using 5% H_2_-95% Ar gas mixture was able to convert 96% of the hematite content in BR into magnetite with the rest converted into metallic iron. Meanwhile the remaining alumina in the residue were converted into water-soluble sodium aluminate solution [15]. Three distinct methods were explored to recover iron and alumina from BR-NaOH pellets through 100% H_2_ reduction at 600 °C, to convert hematite to magnetite (Fe^3+^). In the first process, water leaching and wet magnetic separation were employed, followed by dry magnetic separation, providing an increase in iron recovery from 31.57 wt.% to 38.5 wt.%. The second process involves water leaching followed by smelting of the leach residue to produce sponge iron containing Al and minor Na. The third process involves wet magnetic separation, simultaneous leaching, and a magnetic separation approach, which results in a slight increase in iron content from 31.57 wt.% to 31.85 wt.% in the magnetic fraction and high alumina recovery of 91% [16].

Meanwhile, reduction with H_2_ at high temperature (1000–1200 °C) was able to completely reduce Fe content in BR to metallic Fe [11,12], with results showing retardation of kinetics occurring at T > 1000 °C, suggesting optimum temperature for H_2_ reduction of BR pellets is at 1000 °C [12,13]. The addition of calcite into BR pellets was able to promote the formation of mayenite, a calcium alumino-ferrite phase with high leachability starting from 900 °C and above [17]. However, the recovery of Fe-containing phases from solid-state reduced BR remains an issue due to its physical nature, which exists in miniscule spots with less than 20 µm in particle diameter [12,13]. Carbothermic reduction of BR beyond Fe melting point (>1538 °C) was able to reliably produce pig iron in its own separated phase [8,11]. The remaining issue was recovery of Al content from its slag where almost half of it was trapped in gehlenite (Al_2_Ca_2_O_7_Si) phase, which is difficult to recover via hydrometallurgical means [18].

Previous studies show that the temperature of reduction plays a key role in determining final phase composition and state of reduced BR. Smelting reduction beyond Fe melting point can reliably produce pig iron in separated phase, but it will also produce unleachable slag, which significantly lowers its valorization potential. Meanwhile, heat treating in solid state has its own challenges, which mainly revolves around the separation process of metallic iron that is distributed throughout BR in miniscule spots [19]. Furthermore, in all of the previous studies there is a lack of data related to detail characterization of phases that are expected during reduction and thermal treatment. Thus, the aim of present study is to assess the change in phase compositions of BR and H_2_-reduced BR pellets with increasing temperature through a comparison of experimental data and thermochemistry simulations.

## 2. Materials and Methods

An experimental procedure was designed to study sintering behavior of BR pellets that has been reduced by hydrogen. A mix of BR and Ca(OH)_2_ was made and pelletized, then they were sintered. BR was provided by Mytilineos of Greece (Aluminum of Greece, Athens, Greece) and the calcium hydrate was made by calcination of calcite (CaCO_3_) from Omya S.A. Norway at 950 °C for 2 h followed by hydration by water spraying. The sintered pellets were reduced under different H_2_-H_2_O gas mixtures where H_2_O content was controlled using Cellkraft P-10 humidifier with membrane technology (Cellkraft, Stockhold, Sweden). Bruker D8 ADVANCE DaVinci X-ray diffraction (XRD) and X-ray fluorescence (XRF) (Bruker, Billerica, MA, USA) were employed to analyze phase composition of sintered pellets. Meanwhile, only XRD was employed to analyze phase composition of reduced pellets. XRF analysis was carried out externally by Degerfors Laboratorium. A single pellet from the sintered pellets and reduced pellets was then crushed and sieved under 1 mm for differential thermal analysis (DTA) and thermogravimetric analysis (TGA) to analyze sintering behavior of sintered and reduced pellets with an increasing temperature up to 1400 °C LINSEIS™ TG-DTA equipment (Linseis Messgeraete GmbH, Selb, Germany).

### 2.1. Pelletizing and Sintering

**Figure 1:**
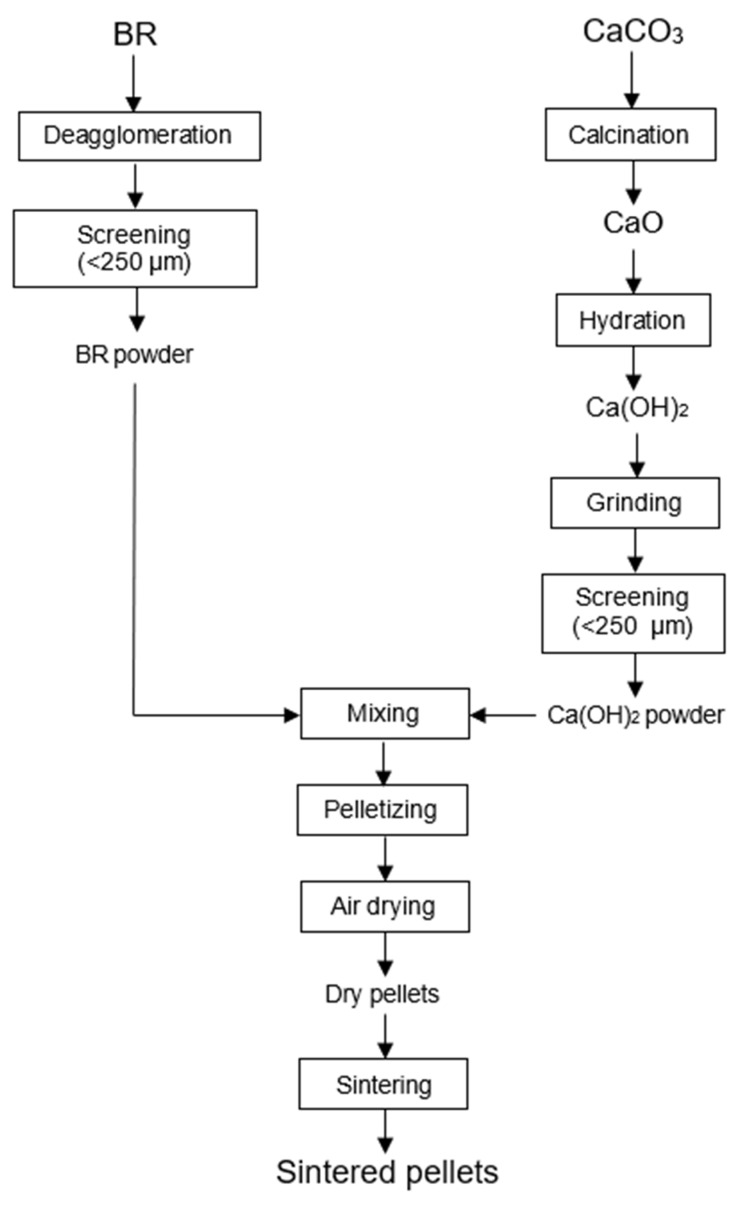
Flowsheet of the pelletizing and sintering process.

BR fines were deagglomerated and screened (<250 µm) to obtain uniform sizing on the mixing process. Raw CaCO_3_ powder was calcined to make CaO powder. Resulting CaO powder was ground and screened (<250 µm) before it was hydrated to make Ca(OH)_2_ powder. BR fines were mixed with Ca(OH)_2_ powder with a mass ratio of 1:0.38, respectively. The ratio was decided based on the stoichiometric ratio of CaO needed to effectively produce calcium aluminate phase in the reduced pellets to allow effective recovery of alumina through leaching. The green pellets were made using a disc pelletizer and screened to obtain pellets with diameter of 4–10 mm, which were then air-dried for 1 day and subsequently sintered at 1150 °C for 2 h in a muffle furnace. The sintered pellets were cooled down naturally inside the furnace for 8 h before being taken out. Flowsheet of the pelletizing and sintering process is shown in Figure 1. Meanwhile, XRF analyses of raw BR, CaCO_3_ powder, and sintered pellet are shown in Table 1, Table 2 and Table 3, respectively.

### 2.2. H_2_ Reduction

**Figure 2:**
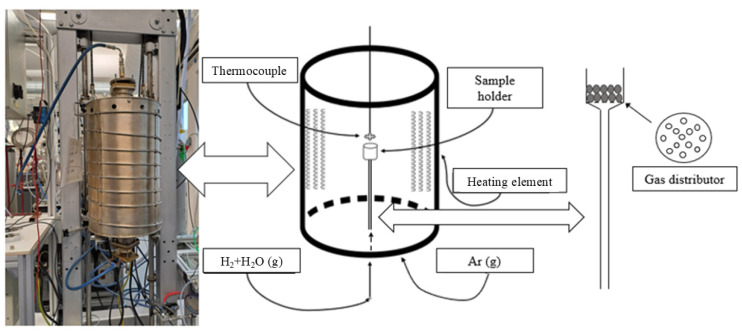
Picture and schematic diagram of reduction furnace and sample holder.

The reduction experiments were conducted using a vertical alumina tube resistance furnace with an outer metallic alloy heating element. Around 20 g of sintered pellet was used in every experiment. Both the furnace and a schematic of its inside are shown in Figure 2. The furnace consists of a cylinder-shaped alumina tube, surrounded by an element that is twined around the furnace. A thermocouple is inserted from the top of the furnace to measure the temperature of the sample holder. The sample holder is made from alumina with gas distributor attached at the bottom to ensure uniform gas distribution to the sample bed. The furnace features a reduction gas inlet positioned at its lower section that goes directly to the sample holder, while the gas, having interacted with the sample, subsequently leaves through the top gas outlet of the furnace. Another gas inlet at a lower section of the furnace, which does not go to the sample holder, is used to flush the furnace with Argon gas at all times of the experiment to prevent water vapor accumulation in the furnace chamber. The heating and cooling were programmed, while the temperature changes were collected by data logging.

**Figure 3:**
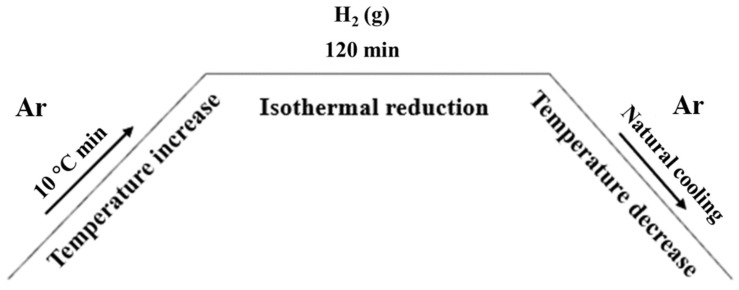
Heating diagram of H_2_ reduction experiment.

H_2_-H_2_O gas mixture composition was controlled by flowing H_2_ gas through a humidifier with set humidity value. The humidifier is a P-10 model made by Cellkraft™ AB with membrane technology. Experiments were performed in 3 different H_2_-H_2_O gas compositions at 600 °C as shown in Table 4. Heating rate of the furnace was 10 °C/minute and held for 2 h at set reduction temperature. Holding time for H_2_ reduction was calculated based on stoichiometric balance of the reaction with at least 300% excess of H_2_ available during every experiment to ensure equilibrium condition was achieved at the end of the experiment. Total flow of H_2_-H_2_O gas mix was kept at 1 L/min in all experiments and the furnace was flushed with 1 L/min of argon at all times, including the cooling and heating period. Schematic heating diagram of the experiment is shown in Figure 3.

### 2.3. Thermal Analysis

To simulate a sintering process during reduction, samples from sintered and reduced BR were subjected to heat treatment up to 1400 °C under inert atmosphere. A single pellet from each hydrogen reduction experiment and the sintered pellet were ground and sieved to <1 mm in preparation for DTA and TGA analysis. Two hundred milligrams of sample were used for every analysis. Both DTA and TGA analysis were carried out at the same time using LINSEIS™ TG-DTA equipment by putting the sample inside an alumina crucible. It is expected that the surface of alumina crucible, which is in contact with the sample, might react with the sample, promoting reactions with alumina as reactant or hindering reactions with alumina as product. However, considering the alumina content in the sample, although quantitative analysis on the result would be inaccurate due to reaction between the sample and the alumina crucible, it should not incur major interference in qualitative analysis, since most of the interference would only happen at the interface are between the sample and the crucible.

The complexity of the chemical composition of BR might mean it is a bigger challenge to reproduce the result obtained in the analyses, especially considering the fact that its production did not undergo any quality control process in any form and its heterogenous nature. However, it is believed that result from the current study can be applied to future studies on bauxite residue with similar chemical content regardless. The analysis was conducted under argon flow of 27.8 cc/min for the whole time, with a heating rate of 20 °C/min up to 1000 °C and 10 °C/min from 1000 °C to 1400 °C. The sample was cooled naturally inside the furnace with argon flow of 27.8 cc/min.

## 3. Results and Discussion

### 3.1. XRD Analysis

**Figure 4:**
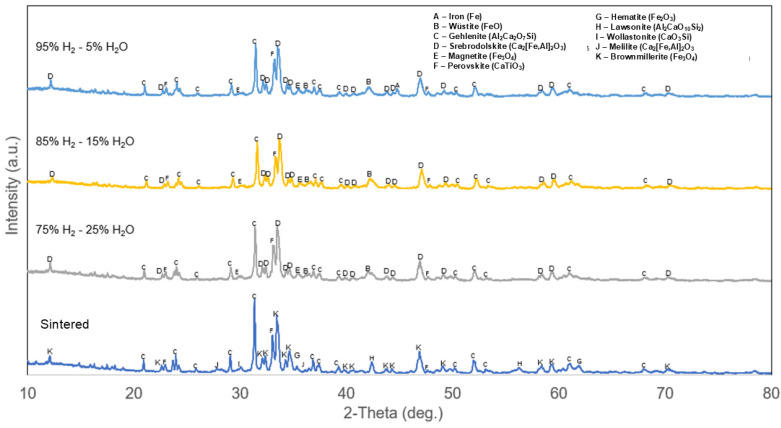
XRD analysis of sintered BR pellets and pellets reduced at 600 °C.

XRD analysis for sintered BR pellets and those reduced at 600 °C is shown in Figure 4. Major phases in the sintered pellets were brownmillerite (Al_0_._441_Ca_2_Fe_1_._559_O_5_), gehlenite (Al_2_Ca_2_O_7_Si), lawsonite (Al_2_CaO_10_Si_2_), wollastonite (CaO_3_Si), melilite (Ca_5_._95_Na_2_._05_O_15_Si_4_), perovskite (CaTiO_3_), and hematite (Fe_2_O_3_). Meanwhile major phases in the reduced pellets were srebrodolskite (Ca_2_[Fe,Al]_2_O_5_), gehlenite (Al_2_Ca_2_O_7_Si), perovskite (CaTiO_3_), magnetite (Fe_3_O_4_), wüstite (FeO), and metallic iron. Brownmillerite and srebrodolskite peaks were in similar positions due to their similar chemical composition and both belong to the brownmillerite subgroup, but with different crystallography. Based on XRD analysis, all the brownmillerite phase elements in sintered pellets were transformed into srebrodolskite during the H_2_ reduction process. It was also observed that most of the calcium silicate-containing phases in the sintered pellets were absorbed into gehlenite during H_2_ reduction, leaving only gehlenite as the only calcium silicate-containing phase in reduced pellets. These results correlate with previous studies, which produced BR pellets with similar phase composition after sintering regardless of slight difference in the production method where calcite (CaCO_3_) was used in the BR pellet mix as opposed to calcium hydrate in the current study; it was also suggested for hydrometallurgical means or smelting process to be employed for industrial valorization of BR [15,20].

By analyzing chemical composition of the phases between sintered pellets and reduced pellets, it can be concluded that only iron oxides were reduced during H_2_ reduction at 600 °C. Magnetite and wüstite peaks were observed in all the reduced pellets, suggesting slow kinetics of the reduction reaction. Meanwhile, metallic iron peaks were observed only in pellets reduced with 95% H_2_-5% H_2_O gas composition, suggesting that the equilibrium for reduction of metallic iron at 600 °C exists between 85% to 95% H_2_ and 5% to 15% H_2_O gas composition.

### 3.2. DTA Analysis

**Figure 5:**
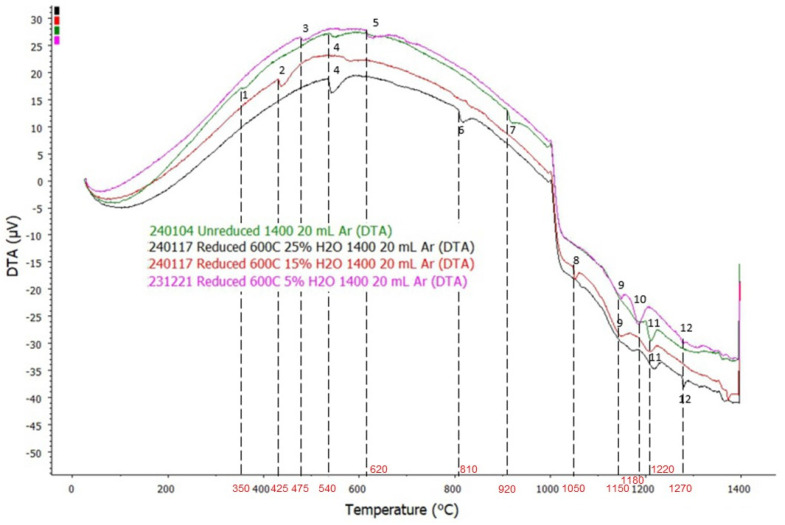
DTA analysis of sintered BR pellets and pellets reduced at 600 °C with temperature points of interest.

DTA analysis for sintered and reduced BR pellets is shown in Figure 5. There are 12 temperature points of interests, which are signified by occurrence of troughs in DTA curves.

Phase equilibrium module of Factsage™ 8.1 was used to analyze and simulate melting process of sintered and reduced pellets. The simulation for sintered pellets was performed based on chemical composition of XRF analysis for sintered pellets, as shown in Table 3, on a basis of 100 g total mass, whereas for simulation of reduced pellets, 10 mol% and 5 mol% of initial hematite composition in sintered pellets were converted into wüstite and magnetite, respectively. The amount of wüstite and magnetite for simulation of reduced pellets was estimated based on XRD result of BR pellets reduced at 600 °C with 95% H_2_-5% H_2_O gas composition. The component input for the simulation of reduced pellets is shown in Table 5.

**Figure 6:**
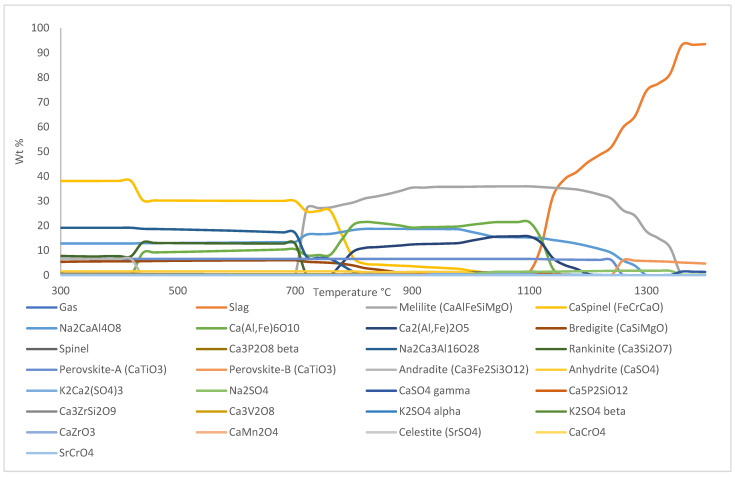
Phase equilibrium simulation of sintered BR pellets from 300 °C to 1400 °C made by Factsage™ 8.1.

**Figure 7:**
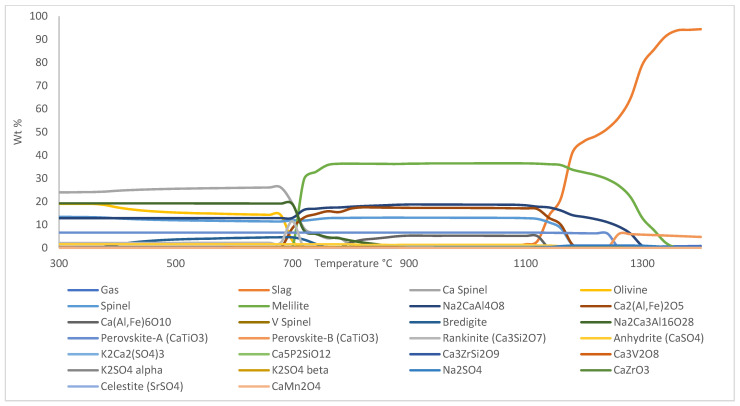
Phase equilibrium simulation of reduced BR pellets from 300 °C to 1400 °C made by Factsage™ 8.1.

The simulation was performed from 300 °C to 1400 °C to investigate the temperature points of interests in the DTA curves. The resulting Factsage™ 8.1 phase equilibrium simulation for sintered pellets is shown in Figure 6 and the result of the simulation for the reduced pellets is shown in Figure 7.

The first temperature point of interest occurred at T ≈ 350 °C, signified by the occurrence of a small trough in the DTA curve of sintered (unreduced) pellet. Based on Factsage™ 8.1 simulation, Ca_5_P_2_SiO_12_ breaks down into tricalcium phosphate-β (Ca_3_P_2_O_8_) and rankinite (Ca_3_Si_2_O_7_), with excess CaO being produced on the first temperature point of interest, which is at T ≈ 350 °C. The reaction that occurred at this temperature is shown in Equation (1).

(1) $$\;2\cdot {Ca}_{5}P_{2}SiO_{12}\;\to 2\cdot {Ca}_{3}P_{2}O_{8}+{Ca}_{3}{Si}_{2}O_{7}+CaO$$

Tricalcium phosphate-β is an unstable phase, and based on the simulation, its reverse reaction occurs at T ≈ 460 °C, which is evidenced by the occurrence of a crest starting from T ≈ 470 °C on the DTA curve of the sintered sample.

The second temperature point of interest occurred at T ≈ 425 °C, where a trough occurred on the DTA curve of the sample reduced with 85% H_2_-15% H_2_O gas composition. Formation of bredigite from part of the olivine phase in the sample is supposed to occur around this temperature based on the simulation of sintered pellets. It is possible that olivine reacted with free CaO in the system to form bredigite with excess magnesium oxide or iron oxide, as shown in Equation (2).

(2) $$\;7\cdot CaO+4\cdot {\left(Mg,Fe\right)}_{2}{SiO}_{4}\;\to \;{Ca}_{7}{Si}_{4}{MgO}_{16}+3\cdot (Mg,Fe)O$$

The third temperature point of interest at T ≈ 475 °C is signified by the occurrence of a trough on the DTA curve of the sample reduced with 95% H_2_-5% H_2_O gas composition. Based on the simulation of reduced pellets, a brief formation of Vanadium-rich spinel occurred at T ≈ 480 °C, however, it immediately breaks at T ≈ 500 °C, which explains the occurrence of a trough at this temperature.

The fourth temperature point of interest at T ≈ 540 °C is signified by the occurrence of a trough on the DTA curve of the sintered sample and the sample reduced with 75% H_2_-25% H_2_O gas composition. There was another nearby trough occurrence as well on the samples reduced with 85% H_2_-15% H_2_O and 95% H_2_-5% H_2_O gas composition, which suggests the same reaction is happening on all samples. Based on the simulation results of both sintered and reduced samples, transformation of potassium sulphate-α (K_2_SO_4_-α) into potassium sulphate-β (K_2_SO_4_-β) occurred around this temperature. The reaction is shown in Equation (3).

(3) $$\;K_{2}{\mathrm{SO}}_{4}‐\alpha \;\to \;K_{2}{\mathrm{SO}}_{4}‐\beta$$

The fifth temperature point of interest is at T ≈ 620 °C, which is signified by the occurrence of a trough on the DTA curve of sample reduced with 95% H_2_-5% H_2_O gas composition. Considering its close proximity to the previous temperature point of interest, the sample is the only one with a significant difference in chemical composition compared to other reduced samples, as it is the only one containing metallic Fe. There is a probability that the same reaction occurred where potassium sulphate-α (K_2_SO_4_-α) transformed into potassium sulphate-β (K_2_SO_4_-β), since there no other change occurred in the simulation around 620 °C.

The sixth temperature point of interest at T ≈ 810 °C is signified by the occurrence of a trough on samples reduced with 75% H_2_-25% H_2_O and 85% H_2_-15% H_2_O gas composition. From the simulation of sintered samples, it was observed that significant formation of mayenite (Ca[Al,Fe]_6_O_10_) and rankinite occurred around this temperature. It is believed that part of a Ca-rich spinel in the sample reacted with andradite (Ca_3_Fe_2_Si_3_O_12_) and CaO in the system to form mayebute and rankinite as shown in Equation (4). This result confirms that the formation of a mayenite phase is thermodynamically possible to induce, starting from 810 °C, as opposed to previous studies that suggest that it starts to occur from 900 °C [15]. The formation of mayenite is highly favorable due to its leachability for the recovery of alumina from BR.

(4) $$\;3\cdot CaO+{CaFe}_{2}O_{4}+\;2\cdot {Ca}_{3}{Fe}_{2}{Si}_{3}O_{12}\;\to \;{Ca(Al,Fe)}_{6}O_{10}\;+\;3\cdot {Ca}_{3}{Si}_{2}O_{7}$$

The seventh temperature point of interest occurred at T ≈ 920 °C, which was signified by the occurrence of a trough on the DTA curve of the sintered sample. Based on the simulation of the sintered sample, celestite (SrSO_4_) and Na_2_CaAl_4_O_8_ start to melt around this temperature to form the slag phase, which may explain the sudden increase in the endothermic nature of the system. It is worth noting that Sr content in the sample is quite low, hence only a few Sr-containing intermediate phases occurred in the simulation at a very low amount.

The eighth temperature point of interest is at T ≈ 1050 °C, which is signified by the occurrence of a trough on the DTA curve of the sample reduced with 85% H_2_-15% H_2_O gas composition. Based on the simulation of reduced pellets, the formation of sodium sulphate (Na_2_SO_4_) is supposed to occur around this temperature. It is proposed that Na_2_CaAl_4_O_8_ in the system breaks down to form slag and reacts with free SO_3_ in the system to form sodium sulphate, as shown in Equation (5).

(5) $$\;{SO}_{3}+{Na}_{2}Ca{Al}_{4}O_{8}\;\to \;\;{Na}_{2}{SO}_{4}+2\cdot {Al}_{2}O_{3}+CaO$$

The ninth temperature point of interest at T ≈ 1150 °C is signified by the occurrence of a trough on the DTA curve of the sample reduced with 85% H_2_-15% H_2_O and 95% H_2_-5% H_2_O gas composition, which is followed by the occurrence of a crest soon after, suggesting an exothermic reaction occurred in the system. Based on the simulation of the reduced sample, a significant amount of calcium alumino-ferrite and brownmillerite (Ca_2_[Al,Fe]_2_O_5_) broke down to form slag around this temperature, which may explain the exothermic nature of the system.

The tenth and eleventh temperature points of interest occur at T ≈ 1180 °C and T ≈ 1220 °C, which is evidenced by the occurrence of troughs on the DTA curve of all the samples. The close proximity between these temperatures suggests that a similar event is happening, with small difference in chemical composition of the sample reduced with 95% H_2_-5% H_2_O due to presence of metallic iron in it causing the event to occur in a slightly lower temperature compared to others. Based on simulations of both sintered and reduced samples, the gas phase starts to occur around these temperatures. The release of gas phase from solid components is generally exothermic, which may explain the formation of troughs in the DTA system.

The twelfth temperature point of interest is at T ≈ 1270 °C, which is signified by the occurrence of a trough on the DTA curve of the sample reduced with 75% H_2_-25% H_2_O and 95% H_2_-5% H_2_O gas composition. Based on the simulation of sintered samples, transformation of perovskite-A into perovskite-B occurred around this temperature, as shown in Equation (6).

(6) $$\;Ca{TiO}_{3}‐A\;\to \;Ca{TiO}_{3}‐B$$

Perovskite-A is a very stable phase that remains relatively unchanged from the beginning up to its transformation into perovskite-B. Meanwhile, perovskite-B is less stable than perovskite-A, and as soon as it forms, it starts to slowly break down into slag phase.

Based on the simulation, most of the events that occurred at each temperature point of interest should apply to all of the samples; however, due to the heterogenous nature of the sample, and the small amount of sample used in the analysis (~200 mg), obtaining uniform distribution of elements in the samples is almost impossible, which explains why some events occurred in one sample but not in the other, despite them showing similar phase composition based on XRD analysis.

### 3.3. TGA Analysis

**Figure 8:**
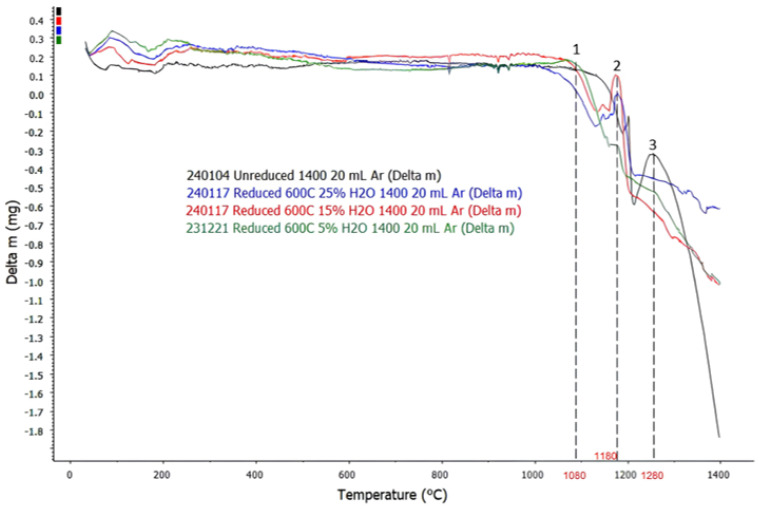
TGA analysis of sintered BR pellets and pellets reduced at 600 °C with temperature points of interest.

TGA analysis for sintered and reduced BR pellets is shown in Figure 8.

The curves in Figure 8 show that there are three temperature points of interests, all of which are signified by significant increases in mass loss rate of the samples. All samples, both sintered and reduced, showed the same trend, where the first significant increase in mass loss rate occurred at T ≈ 1080 °C, followed by the second significant increase in mass loss rate at T ≈ 1180 °C, and the third significant increase in mass loss rate at T ≈ 1280 °C. The same simulation results from Factsage™ 8.1 for sintered and reduced pellets were used to analyze the events happening at the same temperature points of interest.

Based on the simulation results, liquid slag formation starts to occur at T ≈ 900 °C for both sintered and reduced samples; however, the initial amount is really small, since only celestite and small amount of Ca_5_P_2_SiO_12_ form the slag at 900 °C. At this point, the slag consists of mostly CaO, CaS, SrO, and SrS that originated from celestite and Ca_5_P_2_SiO_12_. It is not until T ≈ 1100 °C where the slag starts to pick up iron oxides in the system and increase its amount significantly with increasing temperature. It is proposed that most gases, which are trapped physically in the system, are released during this process, where a larger number of solid phases becoming getting molten, forming the molten slag phase with lower viscosity and less hold-up potential, subsequently causing the first significant increase in mass loss rate of all systems at the first temperature point of interest, which is at T ≈ 1080 °C. Considering the fact that the sintering process generally starts to occur at 0.7 × T_melt,_ it is suggested that partial sintering will start to occur in H_2_-reduced BR pellets from T ≈ 770 °C. Based on this, partial sintering is expected to happen during the H_2_ reduction of BR pellets, which may alter its microstructure, consequently affecting its reactivity and physical properties for further processing.

**Figure 9:**
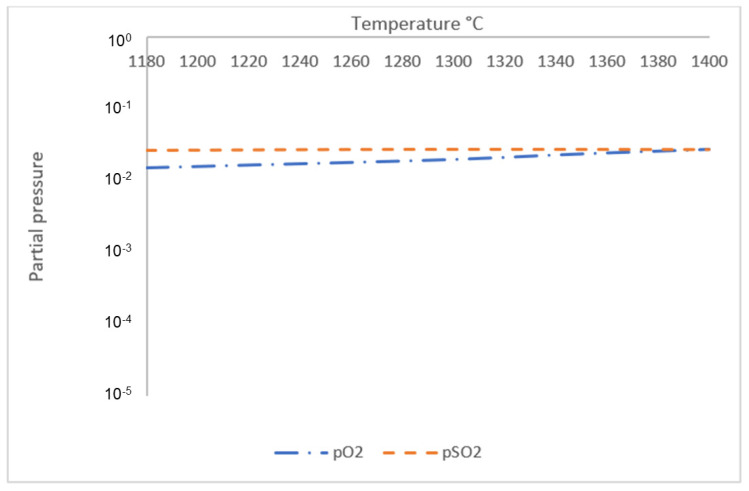
Equilibrium simulation for gas-phase composition of sintered BR pellets from 1180 °C to 1400 °C.

**Figure 10:**
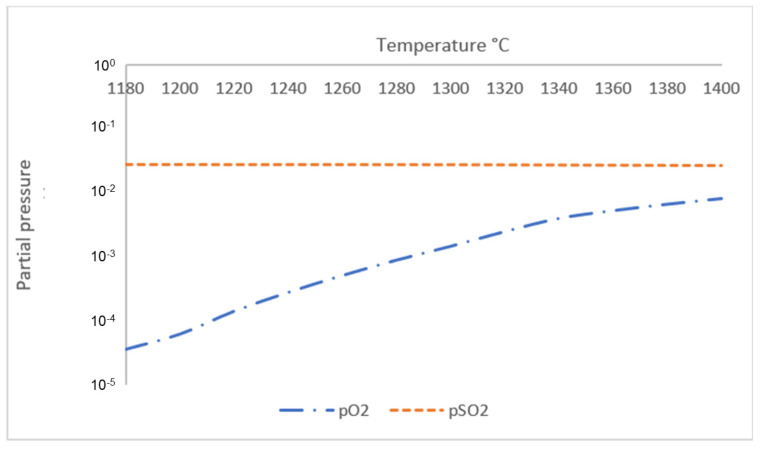
Equilibrium simulation for gas-phase composition of reduced BR pellets from 1180 °C to 1400 °C.

The second temperature point of interest is at T ≈ 1180 °C, which correlates to the DTA curve where a trough followed by a crest occurred at a similar temperature; as mentioned previously, the gas phase starts to from around this temperature. To properly analyze the composition of the gas phase with increasing temperature from 1180 °C to 1400 °C, another simulation was conducted using Factsage™ 8.1, with the addition of 10 liters of argon gas. Similar to the simulation of phase equilibrium, component input for the sintered pellets was performed based on the chemical composition of XRF analysis for sintered pellets as shown in Table 3, on a basis of 100 g total mass, while simulation of the reduced pellets was performed using the component input shown in Table 5. The resulting simulation for the equilibrium of gas-phase composition for sintered pellets is shown in Figure 9 and the result of the simulation for the equilibrium of gas-phase composition for reduced pellets is shown in Figure 10.

Based on the simulation, gas phases that are discharged by both sintered and reduced samples mainly consist of O_2_ and SO_2_ gas, since partial pressure of other gases such as SO, SO_3_, Na_2_SO_4,_ and K_2_SO_4_ are lower by at least two orders compared to either O_2_ or SO_2_. The simulation for the gas-phase composition of the sintered sample suggests that both O_2_ and SO_2_ gas are released at T = 1180 °C. Meanwhile, for the simulation of the gas-phase composition of the reduced sample, the discharged gas consists of mostly SO_2_ at T = 1180 °C, since partial pressure of O_2_ at this temperature is lower by three orders. The difference in gas-phase composition is due to reduced samples having significantly less chemically bonded O_2,_ since more O_2_ has been removed during H_2_ reduction, leaving more chemically bonded SO_2_ to be released into the atmosphere in comparison with O_2_. The issue of SO_2_ emission is a common problem in carbothermic industrial processes, and a similar potential issue might still arise for H_2_-based reduction processes. Many studies have been conducted on methods to reduce SO_2_ emission, such as to utilize BR or limestone slurry as a scrubbing agent [21].

Based on the phase equilibrium simulation, the amount of gas phase in sintered samples is supposed to increase significantly with increasing temperature starting from T ≈ 1280 °C, and for reduced samples, the amount of gas phase starts to increase from T ≈ 1260 °C. In correlation, the simulation result for gas-phase composition of the sintered sample shows that partial pressure of O_2_ starts to increase more significantly with increasing temperature at T = 1300 °C, suggesting that higher reaction kinetics start to force a higher amount of O_2_ gas to be released from the sample, despite oxidative atmosphere, due to existing SO_2_ gas in the atmosphere, which eventually amplifies gas-phase formation with increasing temperature. The simulation result for the reduced sample also shows that the partial pressure of O_2_ is lower by less than one order compared to the partial pressure of SO_2_ at T ≥ 1320 °C, increasing the likelihood for remaining O_2_ in the sample to be discharged into the atmosphere. It is suggested that increased kinetics of gas-phase formation and the release of remaining O_2_ from the sample are followed by an increase in mass loss rate of the system, which explains the third temperature point of interest at T ≈ 1280 °C. Although the lack of real-time phase or gas analysis made it impossible to physically validate the change in phase and gas composition, a common limitation in similar computational studies, resulting simulation values were qualitatively aligned with experimental trends, which justify each other.

## 4. Conclusions

1. Transformation of brownmillerite into srebrodolskite and dissolution of calcium silicate-containing phases into gehlenite occurred during H_2_ reduction of BR pellets.
2. The following reactions were successfully detected during TG–DTA analysis, which was signified by occurrences of crests and troughs in the DTA curves:
  - Ca_5_P_2_SiO_12_ breaks down into tricalcium phosphate-β, rankinite, and CaO at T ≈ 350 °C; however, since tricalcium phosphate-β is an unstable phase, it reverts into Ca_5_P_2_SiO_12_ at T ≈ 460 °C.
  - Formation of bredigite occurred at T ≈ 425 °C, where olivine reacted with CaO to form bredigite. Significant amounts of mayenite and rankinite were formed at T ≈ 810 °C, which originates from the Ca-rich spinel phase in the system. Na_2_CaAl_4_O_8_ in the pellet yields molten slag and reacts with free SO_3_ in the system to form sodium sulphate at T ≈ 1050 °C.
  - Transformation of potassium sulphate-α (K_2_SO_4_-α) into potassium sulphate-β (K_2_SO_4_-β) occurred at T ≈ 540 °C and transformation of perovskite-A into perovskite-B occurred at T ≈ 1270 °C.
3. Formation of liquid (slag and gas) phases were successfully detected during TG–DTA analysis, which was signified by significant increases in the gradients of TGA curves:
  - Initial slag formation for both sintered and reduced pellets occurred at T = 900 °C, with a small liquid slag formation that contains mostly CaO, CaS, SrO, and SrS. The amount of slag phase start to increase significantly with increasing temperature at T ≈ 1100 °C, where it starts to pick up iron oxides in the system, which was then followed by other oxides dissolution/melting in the system.
  - Initial discharge of gas phase from both sintered and reduced pellets occurred at T ≈ 1180 °C, where sintered samples started to release both O_2_ and SO_2_ gas in the system and reduced samples released SO_2_ gas. The amount of gas phase for both samples started to increase significantly with increasing temperature starting from T ≈ 1280 °C, when increased reaction kinetics forces remaining O_2_ in the sample to be released into the atmosphere.
4. Formation of mayenite, which was observed at T ≈ 810 °C, provides an opportunity for alumina recovery from H_2_-reduced BR pellets due to its leachability.
5. Partial sintering is expected to happen during H_2_ reduction of BR pellets starting from T ≈ 770 °C (0.7 × T_melt_), which ultimately may affect reactivity and physical properties of reduced pellets for further processing.

## Figures and Tables

**Table 1:** XRF analysis of raw BR, dry basis (normalized) *.

Composition	Wt%	Composition	Wt%
CaO	9.98	TiO_2_	5.67
MgO	0.26	Na_2_O	3.51
SiO_2_	8.05	K_2_O	0.10
Al_2_O_3_	24.94	P_2_O_5_	0.13
Fe_2_O_3_	46.20	SO_3_	1.07
MnO	0.09	Total	100

* Excluding loss of ignition (LOI).

**Table 2:** XRF analysis of raw CaCO_3_ powder, dry basis (normalized) *.

Composition	Wt%	Composition	Wt%
CaO	98.632	TiO_2_	0.005
MgO	0.547	Na_2_O	0.037
SiO_2_	0.208	K_2_O	0.035
Al_2_O_3_	0.175	P_2_O_5_	0.009
Fe_2_O_3_	0.084	SO_3_	0.263
MnO	0.005	Total	100

* Excluding loss of ignition (LOI).

**Table 3:** XRF analysis of sintered BR pellet.

Composition	Wt%	Composition	Wt%	Composition	Wt%
CaO	29.01	Cr_2_O_3_	0.18	P_2_O_5_	0.12
MgO	0.37	V_2_O_5_	0.15	SO_3_	1.03
SiO_2_	7.66	TiO_2_	3.87	ZrO_2_	0.11
Al_2_O_3_	23.12	NiO	0.06	SrO	0.03
Fe_2_O_3_	30.52	Na_2_O	3.61	Co_3_O_4_	0.02
MnO	0.04	K_2_O	0.10	Total	100

**Table 4:** Experiment conditions for H_2_ reduction.

No.	Gas Composition (vol%)	Reduction Temperature (°C)
1	95% H_2_-5% H_2_O	600
2	85% H_2_-15% H_2_O	600
3	75% H_2_-25% H_2_O	600

**Table 5:** Component input for simulation of reduced BR pellets using Factsage™ 8.1.

Component	Mass (gr)	Component	Mass (gr)	Component	Mass (gr)
CaO	29.01	MnO	0.04	P_2_O_5_	0.12
MgO	0.37	Cr_2_O_3_	0.18	SO_3_	1.03
SiO_2_	7.66	V_2_O_5_	0.15	ZrO_2_	0.11
Al_2_O_3_	23.12	TiO_2_	3.87	SrO	0.03
Fe_2_O_3_	25.94	NiO	0.06	Co_3_O_4_	0.02
Fe_3_O_4_	1.48	Na_2_O	3.61	Total	99.65
FeO	2.75	K_2_O	0.10		

## Data Availability

The original contributions presented in this study are included in the article. Further inquiries can be directed to the corresponding author(s).
